# Draw me a brain: The use of drawing as a tool to examine children's developing knowledge about the “black box”

**DOI:** 10.3389/fpsyg.2022.951784

**Published:** 2022-09-13

**Authors:** Claire Brechet, Nathalie Blanc, Arnaud Mortier, Sandrine Rossi

**Affiliations:** ^1^Univ Paul Valéry Montpellier 3, EPSYLON EA 4556, Montpellier, France; ^2^LPCN - Laboratoire de Psychologie de Caen Normandie, Normandie Univ, UNICAEN, LPCN, Caen, France; ^3^LMNO - Laboratoire de Mathématiques Nicolas Oresme, Normandie Univ, UNICAEN, CNRS, LMNO, Caen, France

**Keywords:** brain, drawing, children, development, representation, neuroeducation

## Abstract

Recent studies in neuroeducation highlight the benefits of teaching children about how the brain works. However, very little is known about children's naive conceptions about the brain. The current study examined these representations, by asking 6–10 year-old children (*N* = 257) and adults (*N* = 38) to draw a brain and the inside of a belly as a control drawing. The drawings were scored using a content analysis and a list of graphic indicators was derived. First, all the graphic indicators used in the brain drawings were different from those used in the belly drawings, suggesting that children are able to distinguish these two organs. Second, with age, children depict (i) an increasing number of indicators, (ii) more complex indicators, (iii) indicators that are more anatomically correct, to depict the brain. There is an important evolution between 6 and 8 years-old but also between 10 years-old and adults. These results are discussed in relation to children's metacognitive knowledge and to their implications for neuroeducation.

## Introduction

What knowledge do children have about the brain as a “black box”? This question is of major interest, particularly with regard to the significance of metacognitive knowledge in school learning. Metacognitive knowledge corresponds to knowledge that a person has of their own cognitive processes and the factors that influence them (Flavell, 1979). Accordingly, having an accurate conception of the brain involves general knowledge about the mental functioning and could promote learning. Like Jolles and Jolles (2021) we defend the idea that it is essential to have knowledge about the brain (structure, function, development) in the same way that it is essential to have knowledge about other organs of the body. The contribution of knowledge we have today on the brain for the improvement of academic learning is no longer to be demonstrated. The American Psychological Association has edited 20 principles from psychological science about effective teaching and learning in preK-12 classrooms, which are for a part of them based on neuroscience literature (American Psychological Association, 2015). This was also supported by the Society for Neuroscience formulating eight essential principles of neuroscience that one should know about the brain and nervous system with educational application from kindergarten to 12th grade (Society for Neuroscience, 2008). The first of these principles is to know the structure and the shape of the brain. The purpose of this study is to interrogate this knowledge through the drawing of the brain. The results obtained could lead to promoting the use of this drawing in class to access the knowledge that students have on the “black box” and thus promote teaching on the role of the brain in school learning. This is in the vein of neuroeducation, an interdisciplinary field of research whose objective is to apply knowledge of brain functioning to classroom practices (Thomas et al., 2019). It has been in full swing for over a decade (Gola et al., 2022), nevertheless, it is surprising how few studies have been conducted on children's developing knowledge about the brain. It seems clear that there is a need to consider children's naive conceptions of the brain particularly in the construction of brain-based educational programs.

The notion of “naive conceptions” corresponds to a way of seeing the world naively or instinctively. As opposed to a scientific conception, it results from intuitive knowledge leading to an understanding of natural phenomena (Vosniadou and Brewer, 1992). Johnson and Wellman (1982) conducted the first study that explored naive conceptions on the relation between mind and brain. Children between the age of 6 and 15 were asked whether various cognitive functions could be possible first without mind and then without brain, or vice versa. With age, children increasingly involve the brain in sensory-motor functions in addition to mental functions. In contrast the mind was dedicated to mental functions. Concerning the mind and brain ontology, although the youngest children did not differentiate between the functions of the mind and the brain, they thought that they were separate in the head. With age, children increasingly believed that the mind depended on the brain. Marshall and Comalli (2012) replicated these results with the same design protocol 30 years later.

Rossi et al. (2015) assessed 8-year-old children's naive mind-brain conceptions, using the Mind-Brain Questionnaire. Children were randomly assigned to one condition: MRI (in which children first participated in a Magnetic Resonance Imaging protocol; Houdé et al., 2011) or control (with no MRI protocol). Children were then presented with a character, placed in different cognitive activities and they had to indicate what the character needed to perform each activity using response cards. This study revealed an educational effect of participation in a MRI protocol on children's naive mind-brain conceptions. Children in the MRI condition seemed to have a better understanding of the relation between mind and brain particularly for dreaming and imagining by materializing the mind into the brain, compared to children in the control condition. Nevertheless, this relation was less clear for seeing, talking, reading, and counting, with no differences between the two conditions. This study emphasizes 8-year-old children's lack of knowledge about the brain and stresses the need to further examine this line of research.

This set of studies could also be linked to what we know about children's thinking abilities through the theory of mind (ToM). The core of this theory, first introduced in 1978 by Premack and Woodruff, relies on the ability to infer mental states of self and others, with many empirical studies showing a progressive shift in children's ability to attribute to others a state of knowledge about a given situation different from their own. As pointed out by Beaudoin et al. (2020), this well-known theory is of interest for many disciplines (e.g., developmental, educational, neuro- and social psychology, social neuroscience). If ToM development during early childhood has highly documented consequences on children's social understanding and social functioning (e.g., Hughes, 2011), its development also has strong intrinsic implications to children's cognitive growth and school readiness (Astington and Pelletier, 2005; Blair and Razza, 2007). According to Wang and Liu (2015), children's mental state understanding is critical to the successful transition to formal schooling, making an integral relation between children's ToM development and their teaching and learning concepts. In line with these studies, it seems relevant to introduce the idea that the way children represent the brain is not unrelated to the way they represent what they know, what they do not know, what others know and what others do not know (e.g., Battistelli and Farneti, 2015). Therefore, in line with the works carried out in neuroeducation that sustain the interest for children to be familiar with the functioning of their brain in order to better grasp learning situations, children's brain conception may be sensitive to ToM development.

Previous studies focus on children's developing conceptualization of brain functions and functioning, but do not address how children portray the brain (i.e., its shape, structure, content, etc.). This issue seems rather complicated to address through verbal methods with children. A body of recent work suggests the use of drawing as an indirect and non-verbal investigation method for this kind of purpose. Indeed, drawing can be reliably used to help children disclose their thoughts on topics that are abstract, not immediately salient in their lives or difficult to talk about (e.g., Ainsworth et al., 2011; Brechet, 2015; Mouratidi et al., 2016). For instance, drawing has been reliably used to examine children's representations of topics such as illness and health (Piko and Bak, 2006; Mouratidi et al., 2016; Bonoti et al., 2019), love (Brechet, 2015), robots (Secim et al., 2021), science (Samaras et al., 2012), death (Bonoti et al., 2013), coronavirus (Bonoti et al., 2022), bullying (Andreou and Bonoti, 2010) and loneliness (Misailidi et al., 2012).

Children's ability to depict the aforementioned topics has been associated with their understanding of the depicted themes but also with their representational drawing skills. When children begin to draw, they first produce traces that are difficult for others to interpret, and then they gradually succeed in producing drawings that are described as “representational” (i.e., depicting elements of reality). Studies on the development of children's representational drawings show a clear age-related improvement between about 3 and 11 years of age (Cox, 2005; Jolley, 2010). Among these representational drawings, the first to appear in children's repertoire is the human figure drawing, which evolves and changes from the age of 3 to about 11. As they become more differentiated in their drawings of the human figure, children also develop graphic models for other themes. Around the age of 5, graphic models for themes such as a house or a tree appear in children's spontaneous drawings. From these early representational drawings, children progress to increasingly visually realistic representations of figures and scenes. Although there are several theoretical approaches in this area, authors generally agree that children's drawing activity is driven by the desire to make realistic representations of the world around them (Luquet, 2001; Willats, 2005). However, after the age of 11, children gradually lose interest in drawing. They begin to consider this as a childish activity (Cox, 2005). As a consequence, many children stop drawing between the age of 10 and 12 and most adults produce drawings similar to those of 12-year-olds (Jolley, 2010).

As a matter of fact, many studies have used drawing to examine how children conceive the human body, by asking them to draw what they think is “inside their bodies” or “inside themselves” (Steward et al., 1982; Eiser and Patterson, 1983; Glaun and Rosenthal, 1987; Reiss and Tunnicliffe, 2001; Reiss et al., 2002; Bartoszeck et al., 2008, 2011; Stears and Dempster, 2017; Andersson et al., 2020). In these studies, the authors examine which body parts and organs are represented by children aged between 4 and 13. This body of research provides information on the proportion of children representing the brain in their drawings (compared to other organs and body parts) by age. The number of drawings depicting a brain increases gradually between the ages of 4 and 7, and from the age of 8, the brain is drawn by at least 80% of children. The brain is consistently drawn after the heart, bones and blood and some studies also show that children draw the brain and belly at about the same age. Although these are valuable data, these studies only report on whether or not children draw the brain but do not provide information about how the brain is depicted when children draw it.

To our knowledge, only two studies have addressed this issue, using the exact same procedure and coding process (Bartoszeck and Bartoszeck, 2012; Jeronen et al., 2016). Precisely, in the most recent study conducted by Jeronen et al. (2016), one classification is used to reveal the conceptions of the brain depicted by Finnish and Brazilian children. This classification comes from the categorization established by Bartoszeck and Bartoszeck (2012) on Brazilian children. In both studies, children aged from 4 to 10 were asked to draw “what they think they have inside their head,” using a pencil. An outline of the head and a portion of the neck were drawn on the blackboard of the classroom to serve as a model. The collected drawings were scored according to the model they related to and classified into one of the 7 following categories: mental image model (i.e., the brain is depicted through mental images), hydraulic model (i.e., the brain is depicted by lines as the flow of a small brook), dog bone model (i.e., the brain is depicted as dog bones all over the skull), enteroid/enteric model (i.e., the brain is depicted by tubes or thick threads similar to the intestine on the top of the skull), epithelial model (i.e., the brain is depicted as patches similar to the epithelial tissues), callote/skullcap model (i.e., the brain is depicted by a callote on the top of the skull) and neuroanatomical model (i.e., the brain is depicted by right and left hemispheres). The results based on these categories indicate that younger children's drawings mostly correspond to the mental image model. As they get older, children start to develop a more morphological representation of the brain. However, the neuroanatomical model is still rarely depicted by 10 years-old children. If these two studies sustain the idea that using a drawing task is a promising method to explore children's conception of the brain, their contribution is mainly qualitative. Indeed, in both studies, the data analysis is only descriptive, with no statistical analysis. Moreover, some drawings are provided as examples to illustrate and support the categorization established, but there is no scoring of the exact content of the drawings. Namely, the specific graphic indicators used by children to depict the brain can only be partly inferred from the description of the models and from the examples of drawings provided.

In the present study, 6–10 year-old children and adults were asked to “draw a brain.” Contrary to the two studies previously mentioned (Bartoszeck and Bartoszeck, 2012; Jeronen et al., 2016), we chose not to give the outline of the head, so as to leave the children free to draw the shape of their choice and to allow us to analyze the shape of the brains drawn too. We chose to start examining children from the age of 6, in order to make sure that they were old enough to both understand the instructions relative to the brain drawing and have the representational graphic skill to depict their ideas (Jolley, 2010). We also chose to limit our research to 10-year olds because previous research suggests that they have more elaborate representations about the brain (Bartoszeck and Bartoszeck, 2012) and because this is the age limit beyond which children tend to stop drawing (Jolley, 2010). Within this age range, we expanded our sample with a group of 8-year-olds, to be able to grasp any change occurring between 6 and 10 years. Also, as previous studies indicated that among older children, only a few of them depicted a brain which was anatomically correct, we decided to complete the sample with a group of adults. Because this was an exploratory study, we could not formulate hypotheses based on the existing literature. However, this study was designed to answer specific research questions. First, how does the depiction of the brain evolve with age? More precisely, whereas previous studies only rated the drawings according to the global model they related to, we chose to use a detailed content analysis to identify what shape and which graphic cues were used to represent the brain and how its graphic representation changed with age. Second, whereas previous studies only asked children to draw a brain, we chose to add a control drawing: children were also asked to produce a drawing representing another part of the body (namely, the inside of a belly), to be compared with the brain drawing. Through this control drawing, we aimed to answer the following question: is the content of brain drawings specific to the brain or can we find similar features in the drawings of another part of the body? Third, as previous research using drawing to examine children's representation of other topics indicated that their drawings reflected their understanding of the depicted themes but also their graphic skills, the whole sample was asked to make two additional drawings, in order to derive an individual measure of graphic level. This was used to answer the following question: does the content of the brain (and control) drawings depend on the participant's graphic level? And fourth, as a complement to the drawings, we also asked children for verbal responses to answer the following question: what do children (and adults) know about the location and functions of the brain, depending on their age, and what are the sources of their knowledge?

## Method

### Participants

There were 295 participants: 257 children aged 6–10 and a group of adults. Children were recruited from elementary schools in the South of France. They were of average socioeconomic background and in their normal school year. Parental written consent was obtained and children were tested in accordance with national and international norms that govern the use of human research participants. Children were divided in three age groups: 6-year-olds (*N* = 76; *M* = 6 years 2 months; *SD* = 8 months; 36 girls), 8-year-olds (*N* = 91; *M* = 8 years 1 month; *SD* = 9 months; 44 girls) and 10-year-olds (*N* = 90; *M* = 10 years 3 months; *SD* = 7 months; 48 girls). The adult group was composed of 38 participants aged 18–45 (*M* = 25 years 2 months; *SD* = 8 years; 21 females). They were university students in arts, humanities or social sciences. They were recruited on campus and voluntarily took part in the study.

### Materials

The materials used for the drawing tasks were white blank A4 paper, an HB pencil, a set of six colored pencils (red, pink, yellow, blue, green, beige), an eraser and a wooden mannequin of a man.

### Procedure

The study was conducted individually in a quiet room in the school and lasted an average of 35 min per participant. First, they were asked to draw a brain and a belly, in a counterbalanced order. For the brain drawing participants were first asked “Do you know where the brain is?” The experimenter noted the answer. If the participant did not know or if the answer was incorrect the experimenter explained “it is an organ that we have in the body, like the heart, but the heart is in here (the experimenter pointed to the location of the heart on her chest) whereas the brain is in the head, here (the experimenter pointed to her head and tapped lightly on her skull).” Then, each participant was instructed to draw a brain: “Here is a blank sheet of paper, a gray pencil and some colored pencils. I would like you to draw a brain.” After the brain drawing, the experimenter asked the participants two additional questions: “What is the brain for?” and “How did you know how to draw it?” The experimenter noted the participants' responses to these questions. For the belly drawing, each participant was asked “Here is a blank sheet of paper, a gray pencil and some colored pencils. I would like you to draw what is inside a belly.” In this study, the belly drawing was designed as a control drawing to be compared with the brain drawing, in order to ensure that children were indeed depicting organ-specific details and not just some random body parts. Our main objective was to assess the representation that the participants have of the brain, thus of one of the organs that exist inside the skull. The choice of the control drawing was conditioned in order to place the participants in a similar condition, i.e., to assess the representation of what exists inside another part of the body. From our point of view, the belly is just as easily identifiable by the participants as the skull, especially for the youngest ones (6 years old). Note that there was no time limit so that the children were free to elaborate the content of their drawings.

Then, participants were asked to produce two additional drawings in order to assess their level of graphic development. They were asked to draw a man running (from a wooden mannequin model) and a house from memory, in counterbalanced order. In the running man drawing task, participants were presented with a model at a distance of about 30 cm and oriented in a profile view with the man running to the right. The participants were encouraged to look carefully at the wooden man and to draw exactly what they saw (but not the base or the pole) including the direction the man was running. In the house drawing task participants were asked to draw a house and to make it look as real and as life-like as they could.

### Coding of the drawings

**Figure 1:**
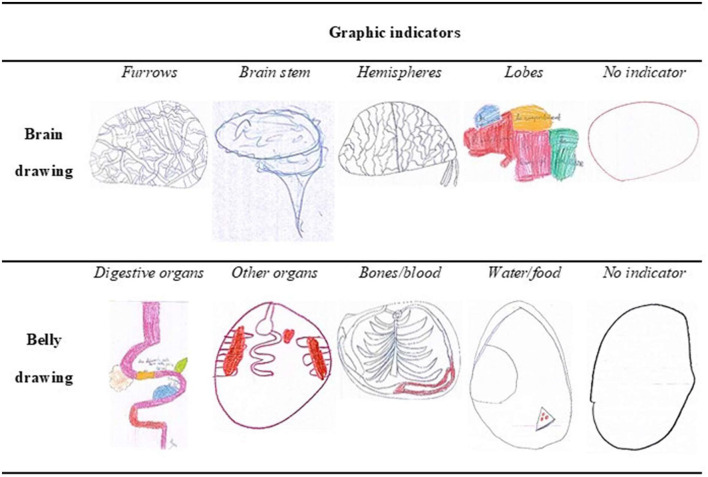
Examples of drawings for each type of graphic indicator relative to the brain and to the belly drawings.

Content analysis was performed to derive the number and types of graphic indicators used to depict the brain and the belly drawings. As our study was the first to examine this question by analyzing the content of the drawings, we did not have access to an existing rating system. We therefore conducted a posteriori analysis, based on the drawings collected, following the basic principles of content analysis (Krippendorf, 1980; Weber, 1990). This scoring process enables to generate a rating system that closely reflects the content of the drawings. It has been extensively used to examine how children depict various kinds of concepts or ideas (e.g., love, coronavirus, health/illness, etc.) through their drawings (e.g., Brechet, 2015; Bonoti et al., 2019, 2022). Based on this method two raters were first asked to independently identify each and every graphic item relative to the brain or the belly in the drawings. The two raters then compared the items they identified and agreed on a final list. Finally, they discussed the items in the list and generated graphic indicators from it (Figure 1).

The following graphic indicators were generated: *furrows, brain stem, hemispheres, lobes* for the brain and *digestive organs, other organs, bones/blood, water/food* for the belly. Figure 1 presents examples of drawings illustrating each indicator.

Subsequently, the raters were asked to independently review and rate each drawing for the presence of any of the four indicators, assigning a single point to each type of graphic indicator included in the drawing. The judges were also given the possibility to categorize drawings as containing no indicator at all. The inter-judge agreements were high (96% for brain drawings and 98% for belly drawings, kappa coefficient = 0.9 for both) and the judges resolved the few cases of disagreement through discussion. Finally, the number of graphic indicators was recorded for each drawing.

**Figure 2:**
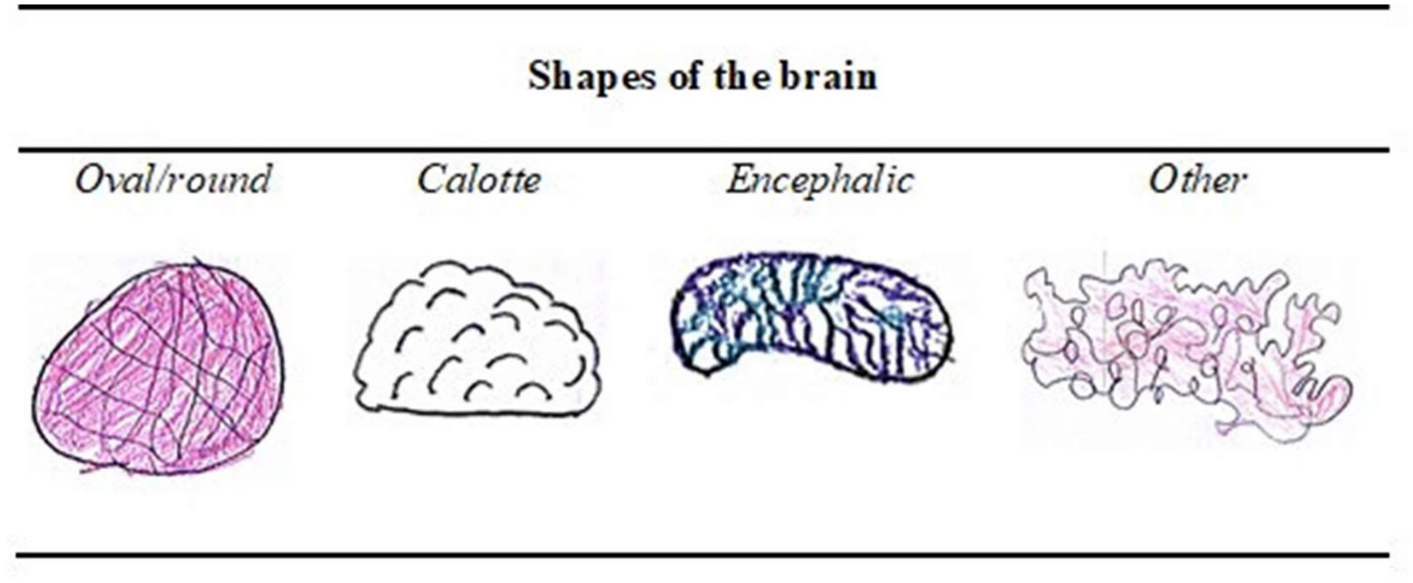
Examples of brain drawings depicting each kind of shape.

The brain drawings were also scored regarding the shape of the depicted brain. To do so, the same two judges were asked to independently identify the different kinds of shapes used to depict the brain. The two raters then agreed on a final list and discussed the exact definition of each shape. The following four kinds of shapes were identified: *round/oval, calotte, encephalic* and *other*. Figure 2 presents examples of brain drawings illustrating each kind of shape. Subsequently, the raters were asked to independently review and rate each brain drawing regarding the depicted shape. The inter-judge agreement was high (90%, kappa coefficient = 0.8) and the judges resolved the few cases of disagreement through discussion.

Following the example of Brechet and Jolley (2014) and Rose et al. (2012), the house drawings and the running man drawings were rated using a revised version of the corresponding scales (respectively, Barrouillet et al., 1994 and Cox et al., 1998). Accordingly, the house drawings were rated on a 13-point scale including the following items: outline of house, roof, roof shape, door, door handle, base of the house, two or more windows, position of windows, proportion of windows, curtains, extraneous items and perspective. The running man drawings were rated on a 23-point scale (with points awarded for direction, overlap, partial occlusion, proportion, detail, recognizability of a person, presence of head, torso, arms, hands, legs and feet, and whether these were depicted as a line or as a zone). Two independent judges performed this scoring and reached an inter-rater agreement of 91% for the house drawings and 93% for the running man drawings (kappa coefficient = 0.8 for both). The cases of disagreement were then discussed and resolved between the two judges. For each participant, the scores on the two drawings were computed as a percentage of the maximum score on each scale. These percentages were then recalculated as scores out of 20 (a number chosen because it was in between the maximum scores of the two scales). Finally, we averaged these two scores to obtain a composite score of graphic development (0–20) for each participant.

### Coding of the verbal responses

As stated above, participants were asked three questions about the brain: one question before they drew, about the location of the brain (“Do you know where the brain is?”) and two questions after they drew, about the function(s) of the brain (“What is the brain for?”) and about the source(s) of their knowledge about the brain (“How did you know how to draw it?”). Their responses to these three questions were coded by two adult judges, using the same procedure as for the coding of the drawings. From the participants' answers, the judges had to classify them thematically, so as to extract the main themes. For the location question, three categories were extracted from the participants' answers: *head, skull* and *I don't know*. For the function question, six categories were identified: *thoughts, intelligence, control, sensory-motricity, life, I don't know*. For the source question, five categories were extracted from the answers: *school, books, television, family*, and *I don't know/I just know it*. Note that the answers to the location question were mutually exclusive: each participant gave only one answer out of the three listed. In contrast, the answers to the function and source questions were not mutually exclusive, as the participants often gave responses relative to more than one category. For example, the following answer to the function question “the brain helps to think, to become smart, it is also used to move and smell, it controls everything that goes on in the body, without it we cannot live” would correspond to the following categories thoughts, intelligence, sensory-motricity, control and life. The judges were then asked to independently review and rate each answer on the basis of the identified categories. The inter-judge agreement was very high (99%) and the judges resolved the few cases of disagreement through discussion.

### Analysis plan

The present study was designed to answer four research questions:

- Q1: How does the depiction of the brain evolve with age?
- Q2: Is the content of brain drawings specific to the brain or can we find similar features in the drawings of another part of the body?
- Q3: Does the content of the brain (and control) drawings depend on the participant's graphic level?
- Q4: What do children (and adults) know about the location and functions of the brain, depending on their age, and what are the sources of their knowledge?

To answer these questions, we examined the number of graphic indicators (in the brain and belly drawings), then the types of graphic indicators (in the brain and belly drawings), the shape of the brain, and finally the responses to verbal questions, as a function of age. First, we carried out a repeated measure analysis of variance (RMANOVA) on the number of indicators (0–4) with Drawing type (Brain, Belly) as a within-participants factor, with Age (6, 8, 10 years-old, adults) as a between-participants factor and with the Level of graphic development as a covariate (Q1, Q2, and Q3). Second, we compared the number of drawings depicting each indicator between each age group using Chi-square analyses for the brain and the belly drawings (Q1 and Q2). For each significant difference we found between age groups, we then decomposed the analysis by examining whether this difference was found for both low and high graphic level subgroups or was specific to one of the subgroups (Q3). And we then repeated the same analysis for the shape of the brain (Q1 and Q3). Finally, we used Chi-square analyses to compare verbal responses relative to the location, the function of the brain and to the source of knowledge, between age groups (Q4).

## Results

### Number of graphic indicators in the brain and in the belly drawings

**Figure 3:**
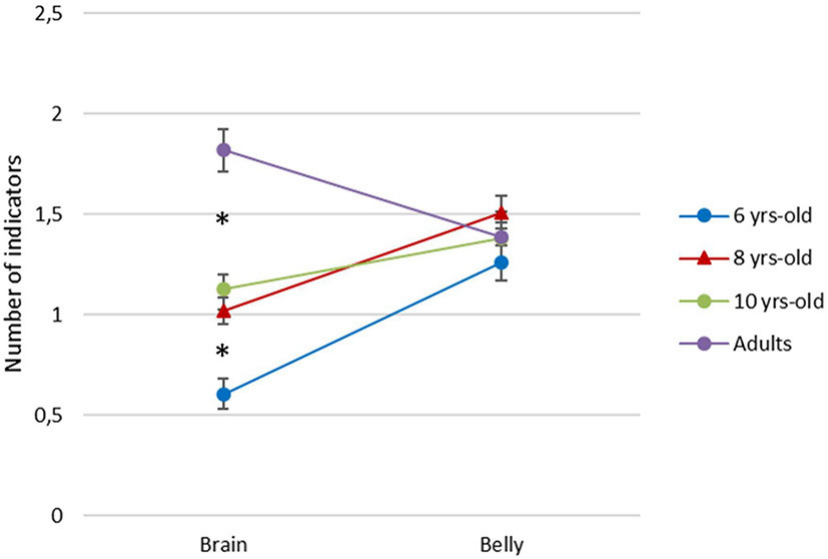
Mean number of graphic indicators as a function of age and drawing type.

Figure 3 presents the mean number of graphic indicators as a function of drawing type and age. We conducted a repeated measure analysis of variance (RMANOVA) on the number of indicators (0-4), with Drawing type (Brain, Belly) as a within-participants factor, with Age (6, 8, 10 years-old, adults) as a between-participants factor, and with the Level of graphic development as a covariate. The results revealed a significant effect of Age [*F*(3,290) = 6.30, *p* = 0.001, η^2^p = 0.061] and Level of graphic development [*F*(1,290) = 7.81, *p* = 0.006, η^2^p = 0.03]. We also found a significant effect of interaction between the Drawing type and the Age [*F*(3,290) = 7.42, *p* = 0.001, η^2^p = 0.07]. Post-hoc comparisons Turkey test revealed a significant increase in the number of graphic indicators for the brain between the age of 6 and 8 (*M*diff = −0.35, *t* = −3.44; *p*bonferroni = 0.01) and between the age of 10 and adulthood (*M*diff = −0.60, *t* = −4.71; *p*bonferroni = 0.001). No significant difference was revealed for the number of graphic indicators for the belly. Moreover, children produced significantly less graphic indicators for the brain than for the belly at 6 years old (*M*diff = −0.61, *t* = −5.32; *p*bonferroni = 0.001) and at 8 years old (*M*diff = −0.47, *t* = −4.78; *p*bonferroni = 0.001).

### Types of graphic indicators in the brain and in the belly drawings

To determine whether participants produced different graphic indicators according to their age we compared the number of drawings depicting each indicator between each age group using Chi-square analyses. We used a Bonferroni correction for multiple comparisons: we divided the standard alpha level of 0.05 by 4 and thus used an adjusted alpha of 0.0125. Considering the results of the previous analysis and to account for the potential effect of graphic skills on these comparisons, the sample was split on the median scores for graphic development (*Median* = 13.98), resulting in two subgroups: low graphic level (*score* <13.98) vs. high graphic level (*score* ≥ 13.98). For each significant difference we found between age groups, we thus decomposed the analysis by examining whether this difference was found for both graphic level subgroups or was specific to one of the subgroups. We have used this categorical approach so that we can present the data in detail by age and graphic level for each indicator. Table 1 presents the number of brain drawings depicting each type of graphic indicator as a function of age and graphic level (low *vs*. high).

**Table 1:** Number (and percentages) of brain drawings depicting each type of graphic indicator as a function of age and graphic level (L1 = low graphic level and L2 = high graphic level).

			**Brain indicators**				
**Age group**	**Graphic level**	***N***	**Furrows**	**Brain stem**	**Hemispheres**	**Lobes**	**No indicator**
6 yrs-old	L1	61	26 (43%)	3 (5%)	2 (3%)	2 (3%)	30 (49%)
	L2	15	12 (80%)	0 (0%)	0 (0%)	1 (7%)	3 (20%)
	Tot	76	38 (50%)	3 (4%)	2 (3%)	3 (4%)	33 (43%)
8 yrs-old	L1	59	38 (64%)	10 (17%)	2 (3%)	9 (15%)	13 (22%)
	L2	32	23 (72%)	5 (16%)	3 (9%)	3 (9%)	7 (22%)
	Tot	91	61 (67%)	15 (16%)	5 (5%)	12 (13%)	20 (22%)
10 yrs-old	L1	28	20 (71%)	2 (7%)	0 (0%)	2 (7%)	5 (18%)
	L2	62	55 (89%)	13 (21%)	7 (11%)	3 (5%)	4 (6%)
	Tot	90	75 (83%)	15 (17%)	7 (8%)	5 (6%)	9 (10%)
Adults	L1	4	4 (100%)	2 (50%)	2 (50%)	0 (0%)	0 (0%)
	L2	34	29 (85%)	7 (21%)	19 (56%)	6 (18%)	0 (0%)
	Tot	38	33 (87%)	9 (24%)	21 (55%)	6 (16%)	0 (0%)
Total	L1	152	88 (58%)	17 (11%)	6 (4%)	13 (9%)	48 (32%)
	L2	143	119 (83%)	25 (17%)	29 (20%)	13 (9%)	14 (10%)
	Tot	295	207 (70%)	42 (14%)	35 (12%)	26 (9%)	62 (21%)

For the brain drawings, the analysis revealed a significant increase in the depiction of *furrows* between 8 (61/91, 67%) and 10 (75/90, 83%) [χ^2^(1) = 6.44, *p* = 0.011]. When decomposing this comparison for both graphic levels, we found a marginal difference only for children with a high graphic level [χ^2^(1) = 4.24, *p* = 0.039]. We also found a significant increase between the age of 6 and 8 in the use of the indicator *brain stem* (respectively, 3/76, 4% and 15/91, 16%) [χ^2^(1) = 6.77, *p* = 0.009]. When running this comparison separately for both graphic levels, we found a marginal difference only for children with a low graphic level [χ^2^(1) = 4.49, *p* = 0.034]. The depiction of *hemispheres* significantly increased between 10-year-olds (7/90, 8%) and adults (21/38, 55%) [χ^2^(1) = 35.25, *p* = 0.001], with a significant difference for low level [χ^2^(1) = 14.93, *p* = 0.001] and high level [χ^2^(1) = 22.11, *p* = 0.001] subgroups. Finally, the number of drawings with *no indicator* significantly decreased between 6 (33/76, 43%) and 8 (20/91, 22%) [χ^2^(1) = 8.79, *p* = 0.003]. When running separate analyses, this difference was significant for children with a low graphic level only [χ^2^(1) = 9.61, *p* = 0.002]. We found no significant age difference in the depiction of lobes.

Table 2 presents the number of belly drawings depicting each type of graphic indicator as a function of age and graphic level. For the belly drawings, the analysis revealed a significant increase in the depiction of *digestive organs* between 6 (41/76, 54%) and 8 (73/91, 80%) [χ^2^(1) = 13.19, *p* = 0.001]. When running this comparison separately for both graphic levels, we found a significant difference only for children with a low graphic level [χ^2^(1) = 14.58, *p* = 0.001]. There was no significant difference with age in the depiction of *other organs, bones*/*blood, water*/*food* and in the number of drawings depicting *no indicator*.

**Table 2:** Number (and percentage) of belly drawings depicting each type of graphic indicator as a function of age and graphic level (L1 = low graphic level and L2 = high graphic level).

			**Belly indicators**				
**Age group**	**Graphic level**	***N***	**Digestive organs**	**Other organs**	**Bones/blood**	**Water/food**	**No indicator**
6 yrs-old	L1	61	28 (46%)	19 (31%)	13 (21%)	11 (18%)	9 (15%)
	L2	15	13 (87%)	7 (47%)	4 (27%)	1 (7%)	0 (0%)
	Tot	76	41 (54%)	26 (34%)	17 (22%)	12 (16%)	9 (12%)
8 yrs-old	L1	59	47 (80%)	22 (37%)	14 (24%)	9 (15%)	6 (10%)
	L2	32	26 (81%)	10 (31%)	5 (16%)	4 (13%)	2 (6%)
	Tot	91	73 (80%)	32 (35%)	19 (21%)	13 (14%)	8 (9%)
10 yrs-old	L1	28	23 (82%)	10 (36%)	3 (11%)	3 (11%)	1 (4%)
	L2	62	60 (97%)	14 (23%)	9 (15%)	2 (3%)	0 (0%)
	Tot	90	83 (92%)	24 (27%)	12 (13%)	5 (6%)	1 (1%)
Adults	L1	4	4 (100%)	2 (50%)	1 (25%)	0 (0%)	0 (0%)
	L2	34	34 (100%)	12 (35%)	0 (0%)	0 (0%)	0 (0%)
	Tot	38	38 (100%)	14 (37%)	1 (3%)	0 (0%)	0 (0%)
Total	L1	152	102 (67%)	53 (35%)	31 (20%)	23 (15%)	16 (11%)
	L2	143	133 (93%)	43 (30%)	18 (13%)	7 (5%)	2 (1%)
	Tot	295	235 (80%)	96 (33%)	49 (17%)	30 (10%)	18 (6%)

To sum up, for the brain drawings, we found a significant increase with age in the depiction of furrows, brain stem and hemispheres and a significant diminution of the number of drawings with no indicator. For the belly drawings, there was a significant increase in the depiction of digestive organs and almost no drawing with no indicator. When considering the participants' graphic level for these comparisons, it appeared that the differences we found between 6 and 8 were related to children with a low graphic level, whereas the differences we found between 8 and 10 and between 10 and adults mainly applied to participants with a high graphic level. A closer look at the indicators depicted at each age suggests that, for the brain drawing, 6-year-olds are divided between those representing furrows (43%) and those depicting no indicators at all (50%). Then, 8- and 10-year-old children can depict furrows and some of them begin to depict brain stem and/or lobes (but some can still produce drawings with no indicators). Finally, adults no longer depict no indicator and can portray furrows, brain stem, lobes, but also hemispheres. To depict a belly, 6 and 8 years-old children can use each of the four indicators: digestive organs, other organs, bones/blood and water/food. At the age of 10, the indicators are quite similar to those depicted by younger children except for water/blood. Finally, adults tend to focus their graphic representation of the belly on digestive organs and some of them also depict other organs.

### Shape of the brain

Table 3 presents the number (and percentage) of drawings using each shape to depict the brain as a function of age and graphic level (low vs. high). We compared the number of drawings depicting each shape between each age group using Chi-square analyses to determine whether participants depicted the brain through different shapes according to their age. We used a Bonferroni correction for multiple comparisons: we divided the standard alpha level of 0.05 by 4 and thus used an adjusted alpha of 0.0125. For each significant difference we found between age groups, we then decomposed the analysis by examining whether this difference was found for both graphic level subgroups or was specific to one of the subgroups.

**Table 3:** Number (and percentage) of drawings using each shape to depict the brain, as a function of age and graphic level (L1 = low graphic level and L2 = high graphic level).

			**Brain shapes**			
**Age group**	**Graphic level**	***N***	**Round/ oval**	**Calotte**	**Encephalic**	**Other**
6 yrs-old	L1	61	41 (67%)	6 (10%)	0 (0%)	14 (23%)
	L2	15	9 (60%)	0 (0%)	1 (7%)	5 (33%)
	Tot	76	50 (66%)	6 (8%)	1 (1%)	19 (25%)
8 yrs-old	L1	59	42 (71%)	6 (10%)	2 (3%)	9 (15%)
	L2	32	17 (53%)	7 (22%)	2 (6%)	6 (19%)
	Tot	91	59 (65%)	13 (14%)	4 (4%)	15 (16%)
10 yrs-old	L1	28	22 (79%)	5 (18%)	0 (0%)	1 (4%)
	L2	62	36 (58%)	11 (18%)	10 (16%)	5 (8%)
	Tot	90	58 (64%)	16 (18%)	10 (11%)	6 (7%)
Adults	L1	4	2 (50%)	0 (0%)	2 (50%)	0 (0%)
	L2	34	12 (35%)	3 (9%)	13 (38%)	6 (18%)
	Tot	38	14 (37%)	3 (8%)	15 (39%)	6 (16%)
Total	L1	152	107 (70%)	17 (11%)	4 (3%)	24 (16%)
	L2	143	74 (52%)	21 (15%)	26 (18%)	22 (15%)
	Tot	295	181 (61%)	38 (13%)	30 (10%)	46 (16%)

The analysis revealed a significant decrease of *round/oval* shaped brains between 10-year-olds (58/90, 64%) and adults (14/38, 37%) [χ^2^(1) = 8.27, *p* = 0.004]. When decomposing this comparison for both graphic levels, we found a marginal difference only for participants with a high graphic level [χ^2^(1) = 4.55, *p* = 0.033]. In contrast, we found a significant increase between the age of 6 and 10 in the depiction of the *encephalic* shape (respectively, 1/76, 1% and 10/90, 11%) [χ^2^(1) = 6.39, *p* = 0.011]. However, this difference was no longer significant when the two graphic levels were considered separately. Adults also drew a significantly higher number of *encephalic* shapes (15/38, 39%) compared to 10-year-olds [χ^2^(1) = 13.68, *p* = 0.001], with a significant difference for participants with a low graphic level [χ^2^(1) = 14.93, *p* = 0.001] and a difference that almost reached significance for participants with a high graphic level [χ^2^(1) = 5.89, *p* = 0.015].

### Verbal responses

Regarding the location question, almost all participants were able to indicate where the brain was located but there was a difference in the words that were used according to age. We used Chi-square analyses to compare responses between age groups. We used a Bonferroni correction for multiple comparisons we divided the standard alpha level of 0.05 by 2 and thus used an adjusted alpha of 0.025. The analyses revealed a significant decline in the use of the word *head* between 10-year-olds (68/90, 76%) and adults (17/38, 45%) [χ^2^(1) = 11.38, *p* = 0.001] and also a marginal decrease in the response *I don't know* between the age of 6 (6/76, 8%) and 8 (1/91, 1%) [χ^2^(1) = 4.76, *p* = 0.029]. In contrast, there was a significant increase in the use of the word *skull* between 6 (6/76, 8%) and 10 (21/90, 23%) [χ^2^(1) = 7.21, *p* = 0.007] and between 10 and adults (21/38, 55%) [χ^2^(1) = 12.36, *p* = 0.001].

For the function question, we first recorded the number of functions cited by each participant. We conducted an analysis of variance (ANOVA) with Age (6, 8, 10 years-old, adults) as a between-participants factor on the number of functions cited. The results revealed a significant effect of Age, *F*(3,291) = 11.64, *p* = 0.001, η^2^*p* = 0.11. A *post-hoc* Tukey test showed that 8-year-olds (*M* = 1.21) cited a higher number of functions than 6-year-olds (*M* = 1.64) (*p* = 0.018). There was no significant difference between the age of 8 and 10 (*M* = 2.00) and between 10-year-olds and adults (*M* = 2.11). Then, to determine whether participants cited different functions according to their age we compared the number of answers corresponding to each function between each age group using Chi-square analyses. We used a Bonferroni correction for multiple comparisons: we divided the standard alpha level of 0.05 by 5 and thus used an adjusted alpha of 0.01. Table 4 presents the number (and percentage) of answers corresponding to each function according to age. The analyses revealed that the number of participants responding *I don't know* significantly decreased between the age of 6 (17/76, 22%) and 8 (5/91, 5%) [χ^2^(1) = 10.31, *p* = 0.001]. The reference to *thoughts* also decreased with age, between 10-year-olds (61/90, 68%) and adults (16/38, 42%) [χ^2^(1) = 7.35, *p* = 0.007]. All the other functions increased with age in the participants' answers. We found a marginal increase in the reference to *intelligence* between 6 (28/76, 37%) and 8 (49/91, 54%) [χ^2^(1) = 4.82, *p* = 0.028]. For *sensory-motricity*, there was also an increase between 6 (5/76, 7%) and 8 (19/91, 21%) [χ^2^(1) = 6.88, *p* = 0.009] and between 8-year-olds and adults (16/38, 42%) [χ^2^(1) = 6.11, *p* = 0.013]. *Life* was more often cited by adults (13/38, 34%) than by 10-year-olds (11/90, 12%) [χ^2^(1) = 8.48, *p* = 0.004]. And there was a difference between each age group in the number of responses relative to *control*: a significant increase between 6 (6/76, 8%) and 8 (21/91, 23%) [χ^2^(1) = 7.04, *p* = 0.008] and between 8 and 10 (41/90, 46%) [χ^2^(1) = 10.15, *p* = 0.001].

**Table 4:** Number (and percentage) of answers corresponding to each function according to age.

		**Functions of the brain**					
**Age group**	***N***	**Thoughts**	**Intelligence**	**Control**	**Sensory-motricity**	**Life**	**Don't know**
6 yrs-old	76	49 (64%)	28 (37%)	6 (8%)	5 (7%)	8 (11%)	17 (22%)
8 yrs-old	91	68 (75%)	49 (54%)	21 (23%)	19 (21%)	6 (7%)	5 (5%)
10 yrs-old	90	61 (68%)	39 (43%)	41 (46%)	28 (31%)	11 (12%)	2 (2%)
Adults	38	16 (42%)	12 (32%)	24 (63%)	16 (42%)	13 (34%)	3 (8%)
Total	295	194 (66%)	128 (43%)	92 (31%)	68 (23%)	38 (13%)	27 (9%)

Regarding the question relative to the sources of participants' knowledge, we compared the number of answers corresponding to each source between each age group using Chi-square analyses. We used a Bonferroni correction for multiple comparisons: we divided the standard alpha level of 0.05 by 4 and thus used an adjusted alpha of 0.0125. Table 5 presents the number (and percentage) of answers corresponding to each source as a function of age. The analyses revealed that the number of participants answering *I don't know* or *I just know it* decreased between the age of 6 (23/76, 30%) and 8 (15/91, 16%) [χ^2^(1) = 4.47, *p* = 0.034]. The reference to *family* significantly decreased with age, between 10-year-olds (16/90, 18%) and adults (0/38, 0%) [χ^2^(1) = 7.72, *p* = 0.005]. In contrast, the reference to *books* significantly increased between 6 (6/76, 8%) and 8 (23/91, 25%) [χ^2^(1) = 8.72, *p* = 0.003]. And for *school*, we found a significant increase between 8 (10/91, 11%) and 10 (28/90, 31%) [χ^2^(1) = 11.05, *p* = 0.001] and between 10-year-olds and adults (27/38, 71%) [χ^2^(1) = 17.39, *p* = 0.001]. Finally, there was no age difference for the source *television*.

**Table 5:** Number (and percentage) of answers corresponding to each source of knowledge as a function of age.

		**Sources of knowledge**				
**Age group**	***N***	**School**	**Books**	**Television**	**Family**	**Don't know**
6 yrs-old	76	4 (5%)	6 (8%)	25 (33%)	19 (25%)	23 (30%)
8 yrs-old	91	10 (11%)	23 (25%)	31 (34%)	17 (19%)	15 (16%)
10 yrs-old	90	28 (31%)	33 (37%)	27 (30%)	16 (18%)	7 (8%)
Adults	38	27 (71%)	8 (21%)	9 (24%)	0 (0%)	3 (8%)
Total	295	69 (23%)	70 (24%)	92 (31%)	52 (18%)	48 (16%)

## Discussion

### How does the depiction of the brain evolve with age?

The main goal of this study was to examine children's developing knowledge about the brain, using drawing as an indirect and non-verbal investigation method. Contrary to previous studies, which only rated the drawings according to the model they related to Bartoszeck and Bartoszeck (2012); Jeronen et al. (2016), we chose to conduct a detailed content analysis of the brain drawings to identify what shape and which graphic cues were used to represent the brain and how its graphic representation changed with age. Our results indicate that, with age, children depict (i) an increasing number of indicators, (ii) more complex indicators, (iii) indicators and shapes that are more anatomically correct, with important shifts between 6 and 8-year-olds but also between 10-year-olds and adults. First, we found a diminution of the number of drawings with no indicator between 6 (48%) and 8 (22%) years-old. And it is worth noting that the number of drawings with no indicator kept decreasing until adulthood. This finding echoes previous studies asking children to draw the inside of their body and showing that the number of drawings depicting a brain increases gradually between the ages of 4 and 7 (Steward et al., 1982; Eiser and Patterson, 1983; Glaun and Rosenthal, 1987; Reiss and Tunnicliffe, 2001; Reiss et al., 2002; Bartoszeck et al., 2008, 2011; Stears and Dempster, 2017; Andersson et al., 2020). In our study, almost half of the 6-year-olds only drew the outline of the brain, as an empty shape. Second, we found a significant increase with age in the depiction of furrows, brain stem and hemispheres, leading to a more anatomically correct representation of the brain. These observations are in line with previous studies showing that the neuroanatomical model of the brain was still rarely depicted by older children (Bartoszeck and Bartoszeck, 2012; Jeronen et al., 2016). The addition of a group of adults in our study enabled us to reveal that the representations kept evolving after the age of 10, in particular with the depiction of hemispheres characterizing the adults' drawings. Finally, we examined the shape of the depicted brains, as a function of age. The results indicate a decrease in the depiction of round/oval shaped brains and an increase in the depiction of the encephalic shape. This evolution matches the one related to the content of the brain, in the sense that the encephalic shape is more likely to contain hemispheres and/or brain stem compared to the round/oval shape.

### Is the content of brain drawings specific to the brain or can we find similar features in the drawings of another part of the body?

In this study, children were also asked to draw a belly, as a control drawing. First, our results indicate that children depicted specific indicators, with no overlap between brain and belly drawings. This suggests that children, even younger ones, do not draw the inside parts of the body all in the same way since distinct indicators were used depending on the part being drawn. This observation supports the validity of the brain drawing. With regard to the belly drawing, there was no overall variation with age in the number of indicators depicted, contrary to the brain drawing. Our results also indicate that at the age of 6 and 8, children produced more indicators in their belly drawing than in their brain drawing. One may conclude that young children have a better representation of the belly. However, when comparing the type of indicators produced in the two drawings, we can notice that the belly drawing is first characterized by a much more “basic” representation, with indicators such as food and water for example. When depicting the brain, children did not use such “basic” indicators. In contrast, either they draw an empty shape, or a shape containing rather relevant and advanced indicators. It is possible that children did not draw basic indicators for the brain simply because there are no such indicators, contrary to the belly. This would lead to an all-or-nothing representation of the brain with either advanced indicators or no indicators at all.

### Does the content of the brain (and control) drawings depend on the participant's graphic level?

Interestingly, when considering the participants' graphic level for the age comparisons we conducted (relative to the types of indicators in the brain and in the belly drawings and also to the shape of the brain), it appeared that the differences we found between 6 and 8 were mostly related to children with a low graphic level. In other words, some indicators or shapes, already represented by young children with a high graphic level, would require a little more developmental progression for children with a low graphic level to represent them in their drawings (e.g., digestive organs in belly drawing). In contrast, the differences we found between 8 and 10 and between 10 and adults mainly applied to participants with a high graphic level. In other words, some indicators or shapes would only shift in number in older participants with a high graphic level (e.g., decrease of the round/oval brain shape, increase of the furrows in the brain drawing). But there were two exceptions to this pattern: the hemispheres indicator and the encephalic shape increased between 10 years old and adults, regardless of the participants' graphic level.

### What do children (and adults) know about the location and functions of the brain, depending on their age, and what are the sources of their knowledge?

Finally, children not only drew a brain but were also asked questions about the location and function of the brain and about the sources of their knowledge. The analysis of the verbal responses provides additional information for interpretation. Regarding the location, almost all participants were able to indicate where the brain was located but there was a difference in the words that were used according to age. There was a decrease of the use of the word “head” and an increase of the use of the word “skull.” This result is not surprising if we consider the age of acquisition of these words reported by Ferrand et al. (2008): for the word “head” the average age of acquisition is 3.92 years while for the word “skull” it is 7.32 years. The subjective frequency of exposure to these words also found in Ferrand et al. (2008) reinforces the relevance to differentiate them, considering that the word “head” is reported to be encountered at least once every 2 days while this frequency falls to once a week for the word “skull.” About the function of the brain, we found an age-related increase in the number of functions that the children cited. While the answers “I don't know” and those referring to thoughts declined, the responses relative to intelligence, sensory-motricity, life and control increased with age. This echoes the developmental pattern we found in children's use of graphic indicators to depict the brain, with the use of indicators which are more complex and anatomically correct with age. This also relates to the gradual disappearance of brain drawings with no indicators. With age, children seem to become aware of the major role that the brain has in driving their behaviors. It remains to be stressed that the vital function of the brain, as an indispensable organ for life, was rare in children but present in adults' responses. On these two aspects, these results show the lesser role granted by young children to this organ which is nevertheless essential to them. Finally, regarding the source of children's knowledge, while the answers “I don't know” or “I just know it” and those referring to the family declined, the responses relative to school and books increased with age. However, it is noteworthy that school does not stand out as a major source of knowledge according to children (5%, 11% and 31% at ages 6, 8, and 10, respectively). From our point of view, there is a need to introduce general knowledge about the brain into school programs, but also to develop students' metacognition in order to help them learning how to learn (Marulis et al., 2020). Lastly, television remained a stable and frequent source cited by all age groups. Obviously, television is still an undeniable source of information for children who can benefit from educational programs, at all ages, with a significant contribution of this medium in the acquisition of knowledge (e.g., Wright et al., 2001). However, this medium can also contribute to the dissemination of neuromyths that are some misconceptions generated by a misunderstanding or a misreading of facts scientifically established by brain research. For instance, the idea that there are critical periods in childhood after which certain things can no longer be learned is such a neuromyth (Dekker et al., 2012). From our point of view, the school should therefore be the major source of information by having teachers trained in brain sciences (Jolles and Jolles, 2021) in order to fight against the dissemination of neuromyths among both students and teachers (Torrijos-Muelas et al., 2021).

### Implications on how knowledge about the brain might be implemented at school and help students to learn

The functioning of the brain is rarely integrated into school curricula and taught from kindergarten to secondary school (Marshall and Comalli, 2012). In France the teaching of the nervous system begins late, i.e., at the age of 12. To compensate for this situation, children implicitly acquire information about the brain through different sources (social environment, exposure to scientific knowledge, media) which could lead them to build an incomplete or erroneous mental representation of the brain, and this seems to reflect our findings. These elements converge toward the idea that it is necessary to instruct children about an organ that they “cannot see” (Society for Neuroscience, 2008; Carew and Magsamen, 2010). Because the brain is what gives children the ability to learn, it is important to teach them what the brain is, what purpose it serves and how they can use it to learn (Lanoë et al., 2015). One way could be to use brain drawing in the classroom to assess students' knowledge (Rossi et al., 2017). This very simple and easy-to-use tool for teachers could be used as a starting point for teaching the role of the brain in academic learning.

However, in order to implement knowledge about the brain in schools it is also necessary to train teachers. As early as 1999, Puckett and collaborators emphasized the promises and the perils of brain developmental research (Puckett et al., 1999). In particular, it is now well-documented that teachers follow to neuromyths (Dekker et al., 2012; Howard-Jones, 2014; Torrijos-Muelas et al., 2021). Because training teachers in educational neuroscience is not enough, exposing them to intuitions and faulty beliefs could be a useful context to give them the tools to deconstruct them. Thus, training in the scientific process and its evaluation would allow them to develop critical thinking skills (Pasquinelli, 2012) in order to resist the seductive look of neuroscientific explanations (Weisberg et al., 2008) and the sirens of popular science journals (Van Atteveldt et al., 2014).

### Limitations

Although informative, this study has several limitations. Regarding the choice of the control drawing, it responded to a number of criteria. First, it was chosen to ensure that the participants were in conditions as similar as possible to those for the brain drawing (i.e., drawing what exists inside another part of the body). Second, we also needed the children to be able to understand from the age of 6 which part of the body was targeted. Finally, although the heart could have also been an interesting choice, we ruled out this option because of the assumption that, at least for the youngest children, we would have obtained a majority of symbolic and not biological drawings. However, it would be interesting for future studies to compare the brain to other body parts or organs in children's drawings to further examine patterns of similarity and difference. Another issue that would have been interesting to address is the orientation of the brain in the drawings: did the participants represent the brain in a frontal, side or top view? As we did not ask the participants to draw the contours of the head, the orientation of the brain was not always clearly identifiable, which therefore did not allow us to present a rigorous analysis on this subject. Nevertheless, among the drawings for which the orientation was identifiable, note that no participant drew a top view of the brain. Instead, the drawings were distributed between side and frontal views. This is an interesting topic because while some indicators seem more representable through a side view (e.g., brain stem), others are more so with a front view (e.g., hemispheres). But did the participants choose an orientation that allowed them to represent the indicators of their choice or did they adapt the drawn indicators to the chosen orientation? This question remains open and would require further investigation. It should be noted, however, that there was not always a straightforward correspondence between orientation and indicators. Indeed, in some drawings the indicators were favored over the realism of the orientation. For instance, hemispheres have often been depicted in brains seen from a side view. Finally, to allow the children to develop the content of their drawings freely there was no time limit and we did not record the time for each drawing. It is possible that the older children, who produced more indicators in their drawings, spent more time drawing. If this was the case, the causal link would still need to be examined since the amount of indicators could be either the cause or the consequence of the amount of time spent drawing.

## Conclusion

In conclusion, this study is part of a long series of research projects that use drawing as a tool for examining children's knowledge, attitudes, and perceptions about events or concepts (e.g., Ainsworth et al., 2011; Brechet, 2015; Mouratidi et al., 2016). Through detailed content analysis of the collected drawings and through the use of additional drawings and questions, we were able to support but also extend the results of previous studies, in order to reach a better understanding of how children conceive the brain. A famous quote from psychologist Ausubel (1968) states that “*the most important single factor influencing learning is what the learner already knows. Ascertain this and teach him accordingly*” (pp. 36). Our results indicate that drawings provide valuable insights into children's current knowledge about the brain that could contribute to the development of effective programs of neuroeducation to improve school-aged children's understanding of how the brain works (Tan and Amiel, 2019; Jolles and Jolles, 2021).

## Data availability statement

The raw data supporting the conclusions of this article will be made available by the authors, without undue reservation.

## Ethics statement

Ethical review and approval was not required for the study on human participants in accordance with the local legislation and institutional requirements. Written informed consent to participate in this study was provided by the participants' legal guardian/next of kin.

## Author contributions

CB is responsible for all aspects of the study including the design, experiment, analysis, and write-up. SR participated to the design of the study, the analysis and to the write-up. AM participated to the analysis and to the write-up. NB participated to the write-up. All authors contributed to the article and approved the submitted version.

## Funding

This work was funded by Plan d'investissement Avenir 100% Inclusion, un Défi, un Territoire. Projet Autorégulation Métacognition Inclusion Scolaire.

## Conflict of interest

The authors declare that the research was conducted in the absence of any commercial or financial relationships that could be construed as a potential conflict of interest.

## Publisher's note

All claims expressed in this article are solely those of the authors and do not necessarily represent those of their affiliated organizations, or those of the publisher, the editors and the reviewers. Any product that may be evaluated in this article, or claim that may be made by its manufacturer, is not guaranteed or endorsed by the publisher.
